# Complete Genome Sequence of a Porcine Kobuvirus Variant Strain from Jiangxi, China

**DOI:** 10.1128/genomeA.01580-16

**Published:** 2017-02-02

**Authors:** Qi Peng, Deping Song, Dongyan Huang, Yanjun Chen, Xinrong Zhou, Fanfan Zhang, Anqi Li, Qiong Wu, Houjun He, Yuxin Tang

**Affiliations:** Department of Preventive Veterinary Medicine, College of Animal Science and Technology, Jiangxi Agricultural University, Nanchang, Jiangxi, China

## Abstract

The complete genome sequence of a porcine kobuvirus (PKoV) variant strain, CH/KB-1/2014 from Jiangxi, China, with a 90-nucleotide deletion in the 2B gene, was determined and characterized. This study provides a better understanding of the molecular characteristics and evolution of PKoV in Jiangxi, China.

## GENOME ANNOUNCEMENT

Porcine kobuvirus (PkoV) is a small, round, nonenveloped, single-stranded, and positive-sense genomic RNA virus in the genus *Kobuvirus*, family *Picornaviridae* (1, 2). The genus *Kobuvirus* comprises three officially recognized species: aichivirus A, aichivirus B, and aichivirus C (1). The genome of PKoV contains a 5′ untranslated region (UTR), a leader (L) protein gene, three structural protein genes (VP0, VP3, and VP1), seven nonstructural protein genes (2A, 2B, 2C, 3A, 3B, 3C, and 3D), and a 3′ UTR (3). A high PKoV infection rate was detected in both diarrheal and asymptomatic piglets in China (4–9).

Small intestinal samples (*n* = 12) were collected from nursing diarrheal piglets in Jiangxi, China, in 2014 to screen for the presence of PKoV with the primer set PKV-S1 and PKV-R1 (10). One of the PKoV-positive samples, designated CH/KB-1/2014, served as the template for amplification of the complete genome with 10 sets of overlapping primers. RNA was extracted from the sample using the TRIzol reagent (TaKaRa, Dalian, China), according to the manufacturer’s instructions. The reverse transcription-PCR (RT-PCR) was carried out based on the standard protocol, and the amplicons were purified using a gel purification kit (TaKaRa) and then cloned into pMD18-T vectors (TaKaRa) for sequencing at both directions. Rapid amplification of 5′ and 3′ cDNA ends (RACE) was performed using a SMARTer RACE 5′/3′ kit (Clontech, Beijing, China) to obtain the extreme ends of PKoV genome. The sequences obtained were assembled and annotated using the SeqMan software (Lasergene 8; DNAStar, Madison, WI).

The full-length genome of CH/KB-1/2014 determined was 8,145 nucleotides (nt) in length, excluding the poly(A) tail, and contained a single open reading frame encoding a polyprotein of 2,459 amino acids (aa), which was slightly shorter than other reference PKoV strains. The genome organization of CH/KB-1/2014 was composed of three structural proteins (VP0, VP3, and VP1) and seven nonstructural proteins (2A, 2B, 2C, 3A, 3B, 3C, and 3D). Notably, CH/KB-1/2014 had a 90-nt deletion in 2B protein region compared with the prototype strain S-1-HUN/2007 (GenBank accession no. EU787450). Phylogenetic analysis of the complete genome sequences revealed that all the Chinese PKoVs were divided into two groups, Chinese group I and Chinese group II. CH/KB-1/2014, along with other 10 Chinese PKoV strains, fell into Chinese group I. Three PKoVs, GS-1/2012/CH (GenBank accession no. KC424639), GS-2/2012/CH (GenBank accession no. KC424640), and K-4/2012/CH (GenBank accession no. KC424638), from Gansu Province in China formed Chinese group II. CH/KB-1/2014 strain had a close phylogenetic relationship with swKoV_CH441, which was isolated from Gansu as well.

A sequence analysis of the VP1 gene of PKoVs indicated that CH/KB-1/2014 shared 81.7 to 87.9% nucleotide (nt) identity and 86.2 to 96.5% amino acid (aa) similarity with other reference PKoVs, respectively. However, CH/KB-1/2014 had low nucleotide and amino acid similarities with both aichivirus A and aichivirus B. CH/KB-1/2014 only showed 59%/50.8% and 51.3%/38.9% nt/aa identities with bovine_kobuvirus (GenBank accession no. NC_004421) and Aichi_virus (GenBank accession no. AB040749), respectively.

In conclusion, the full-length genome sequence of CH/KB-1/2014 was determined and characterized. The study will help understand the epidemiology and evolution of PKoV in Jiangxi, China.

### Accession number(s).

The complete genome sequence of CH/KB-1/2014 was deposited in GenBank under accession number KM051987.

## References

[1] Reuter G, Boros A, Pankovics P 2011 Kobuviruses—a comprehensive review. Rev Med Virol 21:32–41. doi:10.1002/rmv.677. 21294214

[2] Boros Á, Nemes C, Pankovics P, Kapusinszky B, Delwart E, Reuter G 2012 Identification and complete genome characterization of a novel picornavirus in turkey (*Meleagris gallopavo*). J Gen Virol 93:2171–2182. doi:10.1099/vir.0.043224-0. 22875254 PMC3541788

[3] Choi JW, Lee MH, Lee KK, Oem JK 2015 Genetic characteristics of the complete feline kobuvirus genome. Virus Genes 50:52–57. doi:10.1007/s11262-014-1144-y. 25404141

[4] Liu P, Li P, Lyu W, Li X, Li S, Yang F, Huang J, Xu Z, Zhu L 2015 Epidemiological study and variation analysis of the porcine kobuvirus 3D gene in Sichuan Province, China. Virol Sin 30:460–463. doi:10.1007/s12250-015-3632-1. 26637336 PMC8200899

[5] Jin WJ, Yang Z, Zhao ZP, Wang WY, Yang J, Qin AJ, Yang HC 2015 Genetic characterization of porcine kobuvirus variants identified from healthy piglets in China. Infect Genet Evol 35:89–95. doi:10.1016/j.meegid.2015.07.035. 26238210

[6] Yang Z, Jin W, Zhao Z, Lin W, Zhang D, Yu E, Qin A, Yang H 2014 Genetic characterization of porcine kobuvirus and detection of coinfecting pathogens in diarrheic pigs in Jiangsu Province, China. Arch Virol 159:3407–3412. doi:10.1007/s00705-014-2204-2. 25119679

[7] Wang E, Yang B, Liu W, Liu J, Ma X, Lan X 2014 Complete sequencing and phylogenetic analysis of porcine kobuvirus in domestic pigs in northwest China. Arch Virol 159:2533–2535. doi:10.1007/s00705-014-2087-2. 24777826

[8] Wang C, Lan X, Yang B 2016 Molecular epidemiological investigation of porcine kobuvirus and its coinfection rate with PEDV and SaV in northwest China. BioMed Res Int 2016:7590569. 27294133 10.1155/2016/7590569 PMC4884858

[9] Yu JM, Jin M, Zhang Q, Li HY, Li DD, Xu ZQ, Li JS, Cui SX, Yang SH, Liu N, Duan ZJ 2009 Candidate porcine kobuvirus, China. Emerg Infect Dis 15:823–825. doi:10.3201/eid1505.081518. 19402982 PMC2687011

[10] Chen L, Zhu L, Zhou YC, Xu ZW, Guo WZ, Yang WY 2013 Molecular and phylogenetic analysis of the porcine kobuvirus VP1 region using infected pigs from Sichuan Province, China. Virol J 10:281. doi:10.1186/1743-422X-10-281. 24025093 PMC3847588

